# A Rare Congenital Heart Disease in an Elderly Long-Distance Runner: A Case Report

**DOI:** 10.1155/2012/690418

**Published:** 2012-07-19

**Authors:** K. R. Bhamidipati, N. C. Shah, M. C. Connaughton

**Affiliations:** ^1^Department of cardiology, Royal Bournemouth and Christchurch Hospitals, Castle Lane East, Bournemouth BH7 7DW, UK; ^2^St Mary's Hospital, Isle of Wight, Newport PO30 5TG, UK

## Abstract

*Introduction*. Cor triatriatum is a rare congenital heart disease found incidentally in children. Although cor triatriatum can be an incidental finding in asymptomatic adults; it is extremely rare to find elderly patients without symptoms and is unique in a long distance runner. *Case Presentation*. We present the case of an 83-year-old long-distance runner with cor triatriatum sinistrum and atrial fibrillation who continues to be asymptomatic and has continued to run long distances, retaining his excellent functional capacity. *Conclusion*. Cor triatriatum sinistrum is a rare congenital disease, which is often found incidentally in children. Although it is also seen in adults without symptoms at normal exertion, it is rare to have this condition in long-distance runners especially in elderly people with other structural heart disease, that is, atrial fibrillation and mitral regurgitation.

## 1. Introduction

Cor triatriatum is a rare congenital heart disease which forms 0.1%–0.4% of congenital heart disease and affects less than 1 in 200000 people (Office of Rare Diseases, National Institutes of Health, USA). We report the case of an 83-year-old gentleman who was referred to our clinic after a routine ECG showed atrial fibrillation and subsequent workup with echocardiography revealed cor triatriatum sinistrum and mitral regurgitation. He had little by way of symptoms and continued long-distance running without any symptoms. To the best of our knowledge, asymptomatic cor triatriatum in an elderly long-distance runner has never been reported.

## 2. Case Presentation

**Figure 1:**
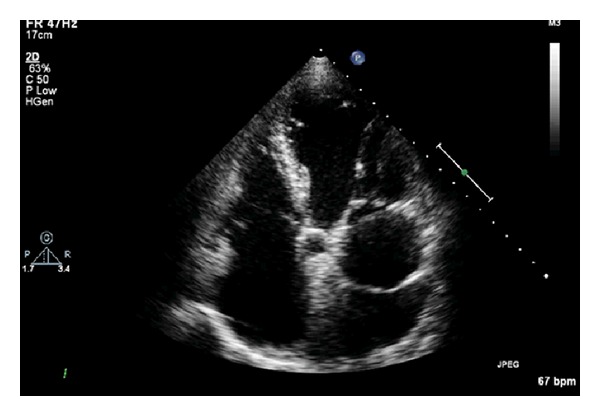
Two-dimensional echocardiographic view (apical four chamber view) showing cor triatriatum sinistrum.

**Figure 2:**
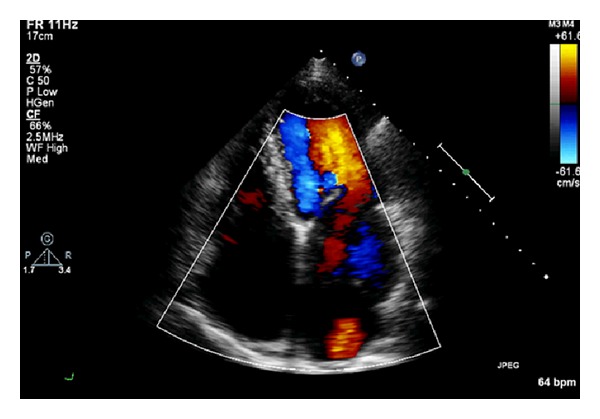
Two-dimensional echocardiographic view (apical 4 chamber view) showing jet of mitral regurgitation.

An 83-year-old gentleman was referred to our cardiology clinic, after atrial fibrillation was diagnosed on a routine physical examination by his general practitioner. ECG confirmed atrial fibrillation, and he was started on digoxin and warfarin by his general practitioner. On direct questioning he denied any symptoms, that is, palpitations, breathlessness, chest pain, or syncope. He was running half marathons without any undue symptoms. He did cut down the distance to about 6 miles recently, but he attributed this to his age and denied any effort-related symptoms. Four years earlier, he had YAG iridotomies for bilateral narrow angle glaucoma. He also had mild cervical kyphosis; for which he had cervical facet joint injections. He also had a basal cell carcinoma excision five years ago. He lived with his wife in a house and was self-caring with excellent mobility. On examination, he had an irregularly irregular pulse consistent with atrial fibrillation, and blood pressure was 110/80 mmHg with no significant difference in the arms. His jugular venous pressure was not elevated, and there was no evidence of pedal oedema, cyanosis, clubbing, icterus, or lymphoedema. There were no radio-radial or radio-femoral delays. Heart sounds were normal with a grade 4 pansystolic murmur best heard at the apex. His chest was clinically clear to auscultation, and examination of his abdomen was within normal limits. All his blood results, including thyroid function tests, were within normal limits.ECG done in clinic showed atrial fibrillation with controlled ventricular rate without any ischaemic changes. Transthoracic echocardiogram revealed a membrane dividing the left atrium into a proximal and a distal segment, that is, Cor triatriatum sinistrum (Figure 1), a moderate jet of central mitral regurgitation (Figure 2) and good left ventricular systolic function. The mitral regurgitation jet passed through the cor triatriatum membrane reaching the distal segment of left atrium. A routine chest X-ray was within normal limits.

His medication included Digoxin 125 mcg OD and Warfarin as guided by INR. As the patient was asymptomatic despite running long distances, it was felt that further investigation or consideration of surgical treatment would be superfluous. He has been reviewed periodically in outpatient clinic, and he continues to run long distances without symptoms.

## 3. Conclusion

Cor triatriatum is a very rare congenital heart disease in adults. Usually it is associated with symptoms, but it is not uncommon to find patients without symptoms. Our patient has continued to run long distances without symptoms despite his cor triatriatum and atrial fibrillation. To our knowledge, he is the oldest patient reported with this condition, and this case report is the only one reported in long-distance runners without symptoms.

## 4. Discussion

Cor triatriatum, which was first reported in 1868, is an extremely rare disease that affects 1 in 200000 people (as per the Office of Rare Diseases of the National Institute of Health, USA) and forms about 0.1% to 0.4% of congenital heart disease [1]. As the name suggests, it gives the impression of having three atria but is due to the presence of a fibromuscular membrane across the atria. This membrane could be total or partial with a perforation. The first variety is associated with predominantly paediatric population who are symptomatic and need surgical correction. The second variety is usually seen in adults and does not usually need surgery. Cor triatriatum is commonly seen in the left atrium, which is called cor triatriatum sinistrum and is rarely seen in the right atrium called cor triatriatum dextrum. In the sinister variety, the proximal chamber receives drainage from the pulmonary veins. In Dexter variety,the proximal chamber receives the venous blood from the venae cavae, and the distal chamber is in contact with the tricuspid valve. There are 3 theories explaining the cause for the formation of cor triatriatum. First theory is the “Malincorporation theory” [2], in which the common pulmonary vein fails to direct the pulmonary circulation into the left atrium, and the common pulmonary venous ostium remains narrow, creating a narrow band like membrane. However, this theory fails to explain the presence of fossa ovalis. The “Malseptation theory” [3] proposes that the membrane is an abnormal growth of the septum primum. The “Entrapment theory” [4] attributes it to the entrapment of the common pulmonary vein by the sinus venosus. Cor Triatriatum can be associated with other cardiac anomalies like atrial septal defects, mitral regurgitation, persistent foramen ovale, and persistent superior vena cava. Patients could be asymptomatic [5] or may present with a host of symptoms [6], that is, dyspnoea, effort intolerance, palpitations, haemoptysis, and systemic embolism. Echocardiography is the investigation of choice and is often enough to delineate the size and extent of the defect. Right heart catheterisation may reveal raised right atrial, right ventricular, pulmonary artery, and pulmonary capillary wedge pressure and help to confirm the diagnosis. CT chest can be used to confirm the diagnosis. ECG is nonspecific and may show signs of right heart strain. Surgical correction is required only in symptomatic patients [7, 8] and usually involves complete resection of the membrane and correction of any associated defects, that is, closure of atrial septal defect and so forth. Medical treatment involves controlling ventricular rates and anticoagulation to prevent systemic embolisation in patients with atrial fibrillation and decongestive treatment with diuretics and vasodilators in those with signs of fluid overload. Inotropic agents are rarely required in patients during perioperative period.

## Abbreviations

YAG: Yttrium aluminium garnet
CT: Computerised tomography
ECG: Electrocardiogram.
